# Community Service Utilization and Aging Attitudes in Older Chinese Adults: The Role of Chronic Conditions

**DOI:** 10.1093/geroni/igaf122.3902

**Published:** 2025-12-31

**Authors:** Zhijing Xu, Yaguang Zheng, Bei Wu

**Affiliations:** New York University, New York, New York, United States; New York University, New York, New York, United States; NYU Shanghai, Shanghai, China (People’s Republic)

**Keywords:** Community Service Utilization, Aging Attitudes, Chronic Conditions

## Abstract

Abstract As China faces an aging population and rising chronic disease prevalence, understanding factors shaping aging attitudes is essential. This study investigates how community service utilization was associated with aging attitudes among older Chinese adults and whether having chronic conditions made a difference. Data from the 2020 China Longitudinal Aging Social Survey (CLASS) were analyzed using multiple regression. Among 7,111 participants aged 60 and older, 5,574 (78.38%) had chronic conditions. Among individuals with chronic conditions, home visits were significantly associated with more negative attitudes toward aging (b = -1.241, p < 0.001), while services such as accompaniment to medical appointments (b = 1.185, p = 0.026), household assistance (b = 0.628, p = 0.033), and participation in daycare center activities (b = 1.279, p = 0.006) were associated with more positive attitudes toward aging. Among older adults without chronic diseases, elderly meal delivery was the only service significantly associated with more negative attitudes toward aging (b = -3.065, p = 0.048). In conclusion, community service utilization plays a significant role in shaping aging attitudes, with varying effects depending on the presence of chronic conditions. Services that promote independence, such as accompaniment and household assistance, are more likely to foster positive aging perceptions, whereas services like home visits and meal delivery may reinforce negative views, especially among those with chronic diseases.

